# *Paenibacillus larvae* Bacteremia in Injection Drug Users

**DOI:** 10.3201/eid1603.091457

**Published:** 2010-03

**Authors:** Siegbert Rieg, Tilman Martin Bauer, Gabriele Peyerl-Hoffmann, Jürgen Held, Wolfgang Ritter, Dirk Wagner, Winfried Vinzenz Kern, Annerose Serr

**Affiliations:** University Hospital, Freiburg, Germany (S. Rieg, T.M. Bauer, G. Peyerl-Hoffman, J. Held, D. Wagner, W.V. Kern, A. Serr); Reference Laboratory of the World Organisation for Animal Health, Freiburg (W. Ritter)

**Keywords:** Paenibacillus larvae, bacteremia, bacteria, intravenous drug abuse, honey, methadone substitution, American foulbrood, dispatch

## Abstract

*Paenibacillus larvae* causes American foulbrood in honey bees. We describe *P. larvae* bacteremia in 5 injection drug users who had self-injected honey-prepared methadone proven to contain *P. larvae* spores. That such preparations may be contaminated with spores of this organism is not well known among pharmacists, physicians, and addicts.

As a consequence of needle sharing and repeated parenteral administration of nonsterile material, injection drug users risk becoming ill from a variety of infections, including HIV, hepatitis C, endocarditis, and skin and soft tissue infections (*1*). Febrile episodes in injection drug users are common, yet distinguishing between febrile reactions caused by toxins or impurities in the injected substance and true infections may be difficult (*2*,*3*). Methadone hydrochloride, which is widely used for opioid substitution, can be mixed with viscous substances such as syrup to yield a solution that is not suitable for misuse through self-injection. Methadone syrup is intended to be taken only as an oral medication. Some pharmacies use honey instead of syrup to prepare such a solution.

*Paenibacillus larvae* is a spore-forming gram-positive microorganism known for its ability to cause American foulbrood, a severe and notifiable disease of honey bees (*Apis mellifera*) (*4*) (Figure). *P. larvae* is endemic to bee colonies worldwide. The organism can be cultured from <10% of honey samples from Germany but from >90% of samples from honeys imported from other countries (*5*). *P. larvae* spores are highly resilient and can survive in honey for years (*6*,*7*). We describe *P. larvae* bacteremia in 5 patients who had a history of intravenous drug abuse and were in a program of opioid substitution that used methadone.

**Figure:**
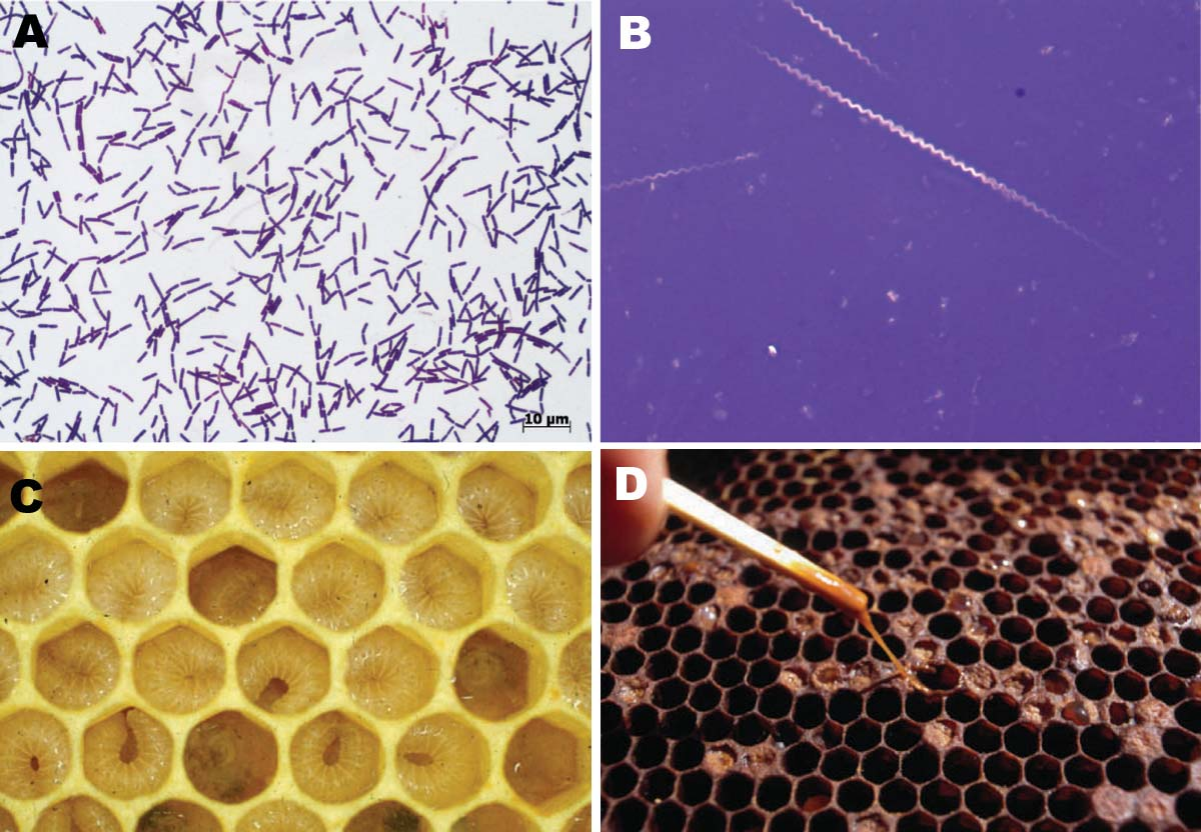
*Paenibacillus larvae* gram-positive, spore-forming, rod-shaped bacteria (A) (Gram stain, original magnification ×1,000) with the ability to form giant whips upon sporulation (B) (nigrosine stain, original magnification ×1,000). In American foulbrood (AFB), newly hatched honey bee larvae become infected through ingestion of brood honey containing *P. larvae* spores. After germination and multiplication, infected bee larvae die within a few days and are decomposed to a ropy mass, which releases millions of infective spores after desiccation. C) AFB-diseased larvae are beige or brown in color and have diminished segmentation (healthy and AFB-diseased larvae). D) Clinical diagnosis of AFB can be made by a matchstick test, demonstrating the viscous, glue-like larval remains adhering to the cell wall.

## The Study

All patients sought treatment for fever ranging from 37.8°C to 39.8°C and admitted to continuing to inject illicit drugs or methadone. Information about patient characteristics, clinical signs and symptoms, laboratory and micobiologic investigations, and treatment details are summarized in the Table. *P. larvae* was identified in blood cultures (BacT/ALERT 3D-System; bioMérieux, Marcy l’Etoile, France) of each patient described. The clinical course of *P. larvae* bacteremia was benign in 3 patients, and complications developed in 2 patients. Patient 1 had relapsing disease and spontaneous bacterial peritonitis; patient 4 had pulmonary embolism without definite evidence of septic embolism. Patients 2 and 3 recovered without specific antimicrobial drug treatment; for patients 4 and 5, defervescence and negative follow-up blood cultures were observed after they received treatment with β-lactam agents (imipenem or cefuroxime). The recurrent *P. larvae* infection observed in patient 1 was probably the consequence of repeated injection of contaminated methadone rather than an inadequate response to antimicrobial drug therapy.

**Table:** Patient characteristics, clinical presentation, treatment, and laboratory and microbiologic results of 5 patients with *Paenibacillus larvae* bacteremia*

Characteristic	Patient no.				
	1	2	3	4	5
Age, y/sex	28/F	32/M	20/M	35/M	27/F
Date evaluated	2003 Jul	2003 Sep	2003 Oct	2004 Feb	2008 May
Clinical samples with identification of *P. larvae*†	Culture of ascites (2003 Jul), blood culture (2003 Aug)	Blood culture	Blood culture	Blood culture	Blood culture
CRP, mg/L	43	17	11	37	40
Leukocyte count, × 10^9^/L	23.0	13.0	9.3	11.8	19.2
Medical history	IVDA, hepatitis C, Child B liver cirrhosis with refractory ascites	IVDA, hepatitis C, hepatitis B	IVDA, hepatitis C	IVDA, hepatitis C, history of hepatitis A	IVDA, hepatitis C, alcohol abuse
Clinical signs and symptoms	Decompensated liver cirrhosis, ascites, fever (39.2°C)	Persistent weakness and malaise, fever (39.2°C)	Somnolence, fever (38.2°C)	Tachypnoe, right-sided pleuritic chest pain, fever (37.8°C)	Severe anemia, spontaneous mucosal bleeding, fever (39.8°C)
Clinical conditions other than bacteremia	Bacterial peritonitis, hepatic encephalopathy after TIPS placement	Acute hepatitis B diagnosed 1 mo before bacteremia, eosinophilia	Methadone/ diazepam overdose	Pulmonary embolism, infarction pneumonia, deep vein thrombosis	Subsequently diagnosed with ITP, *Paracoccus yeei* and *Micrococcus luteus* bacteremia
Treatment (duration)	Meropenem (7 d) followed by ampicillin IV (2 d), then meropenem (7 d) followed by penicillin G (14 d)	None	None	Cefuroxim IV (7 d)	Imipenem (21 d)

*CRP, C-reactive protein (reference range <5 mg/L); IVDA, intravenous drug abuse; TIPS, transjugular intrahepatic portosystemic shunt; ITP, idiopathic thrombocytopenic purpura; IV, intravenous. †*P. larvae* identified after culture using 16S rRNA gene sequencing.

In 2 cases, culture of the honey used to prepare methadone or of honey-containing ready-to-use methadone also yielded *P. larvae*. Honey and methadone samples were diluted in sterile phosphate-buffered saline and cultured with or without heat pretreatment (90°C, 10 min) under aerobic and anaerobic conditions at 37°C for 3–4 days by using Columbia blood agar and MYPGD (Mueller-Hinton broth, yeast extract, potassium phosphate, glucose, pyruvate) agar. Colonies from positive blood or honey or methadone cultures with an appropriate macroscopic appearance and gram-stain morphology as well as negative catalase reaction were further identified by PCR amplification and 16S rRNA gene sequencing according to published protocols (*8*). Obtained sequences were analyzed by using the BLAST algorithm (http://blast.ncbi.nlm.nih.gov/Blast.cgi).

## Conclusions

We detected *P. larvae* in sterile compartments of 5 patients with clinical and laboratory evidence of infection. Given the fact that all patients were injection drug users, the mode of infection was thought to be intravenous administration of contaminated methadone, resulting in *P. larvae* bacteremia. Our hypothesis is supported by the isolation of *P. larvae* from honey or honey-containing methadone provided to 2 patients.

Recently, several *Paenibacillus* species have been reported to cause bacteremic infections in humans. Among these are *P. thiaminolyticus* (bacteremia in a patient undergoing hemodialysis) (*9*), *P. konsidensis* (bacteremia in a febrile patient with hematemesis) (*10*), *P. alvei* (prosthetic joint infection with bacteremia) (*11*), and *P. polymyxa* (bacteremia in a patient with cerebral infarction) (*12*). Furthermore, the novel species *P. massiliensis*, *P. sanguinis,* and *P. timonensis* were isolated from blood cultures of patients with carcinoma, interstitial nephropathy, and leukemia, respectively (*13*). Pseudobacteremia of *P. hongkongensis* and *P. macerans* has been reported (*14*,*15*).

Several aspects provide strong evidence for a genuine *P. larvae* bacteremia in the cases described here. First, the present cases were observed over a period of several years, and detection of *P. larvae* thus occurred in different charges of blood culture bottles, which argues against pseudobacteremia. Second, isolation of *P. larvae* was reported independently by 2 microbiology laboratories, making contamination highly unlikely. Third, in patient 1 isolation succeeded at different times and in samples of different compartments. Moreover, the detection of *P. larvae* in honey-prepared methadone and honey strongly suggests genuine bacteremia as a consequence of injection of contaminated material.

Biochemical and molecular identification of *P. larvae* may be difficult and time-consuming. Misinterpretation of blood culture results because of incomplete differentiation or confusion with other gram-positive spore forming-bacteria (e.g., *Bacillus* species) has to be taken into consideration. Underestimation of the frequency of true *P. larvae* bacteremia therefore cannot be excluded. Thus, infectious disease physicians, microbiologists, and pharmacists need to be aware that injection of material contaminated with *P. larvae*, such as honey-prepared methadone, may cause bacteremic infection.
